# Research status and challenges of *Mycoplasma pneumoniae* pneumonia in children: A bibliometric and visualization analysis from 2011 to 2023

**DOI:** 10.1097/MD.0000000000037521

**Published:** 2024-03-15

**Authors:** Congcong Liu, Rui Wang, Shuyi Ge, Binding Wang, Siman Li, Bohua Yan

**Affiliations:** a Hospital of Chengdu University of Traditional Chinese Medicine, Chengdu, China.

**Keywords:** bibliometric analysis, children, CiteSpace, *Mycoplasma pneumoniae* pneumonia, VOSviewer

## Abstract

**Background:**

*Mycoplasma pneumoniae* (MP) infections occur in regional outbreaks every 3 to 7 years, lasting up to 2 years. Since this fall, there has been a significant rise in MP infections among children in China, indicating a regional epidemiological trend that imposes an increased national public health burden. To date, bibliometric methods have not been applied to studies on MP infection in children.

**Methods:**

We searched for all relevant English publications on MP pneumonia in children published from 2011 to 2023 using Web of Science. Analytical software tools such as Citespace and VOSviewer were employed to analyze the collected literature.

**Results:**

993 articles on MP pneumonia in children were published in 338 academic journals by 5062 authors affiliated with 1381 institutions across 75 countries/regions. China led in global productivity with 56.19%. Among the top 10 prolific organizations, 8 were Chinese institutions, with Soochow University being the most active, followed by Capital Medical University and Zhejiang University. Zhimin Chen from Zhejiang University School of Medicine exhibited the highest H-index of 32. Keyword co-occurrence network analysis revealed 7 highly relevant clusters.

**Conclusion:**

The current research hotspots and frontiers in this field are primarily MP pneumonia, refractory MP pneumonia, lactate dehydrogenase, asthma, and biomarker. We anticipate that this work will provide novel insights for advancing scientific exploration and the clinical application of MP pneumonia in children.

## 1. Introduction

*Mycoplasma pneumoniae*(MP) stands out as a common culprit behind both upper and lower respiratory tract infections in humans.^[1]^ In children, MP is responsible for 10% to 40% of cases of community-acquired pneumonia (CAP), a figure that can escalate to 20% to 70% during pandemics.^[2–4]^A decade-long national surveillance of acute respiratory infections indicates that MP exhibits the highest detection rate among atypical pathogens in Chinese children aged 5 to 7.^[5]^ MPP, resulting from MP, is a prevalent respiratory infection in children.

MP infections manifest in regional outbreaks every 3 to 7 years, persisting up to 2 years. Notably, China has witnessed a nationwide surge in pediatric mycoplasma infections, particularly during the fall and winter months. While MPP is typically self-limiting with mild symptoms such as fever, cough, and dyspnea, it can lead to pulmonary complications like occlusive bronchitis, bronchiectasis, acute respiratory distress syndrome (ARDS), and necrotizing pneumonia. Moreover, an array of extrapulmonary manifestations, including pulmonary embolism, Stevens-Johnson syndrome, cardiomyopathy, and meningitis, is on the rise.^[6,7]^

Bibliometric analysis serves as a valuable tool for understanding productivity levels, publication trends, and features by providing statistical descriptions of publications.^[8]^ Widely-used bibliometric applications such as CiteSpace^[9]^ and VOSviewer^[10]^ are commonly utilized to depict literature review findings, extensively applied in the medical sector. Remarkably, bibliometric methods have not been applied to studies of pediatric mycoplasma pneumonia. Nevertheless, clarity is still needed on the mechanism of pediatric mycoplasma pneumonia, with diagnostic methods varying among healthcare providers. Optimal treatment guidelines have yet to be established, and further knowledge must be gleaned from pertinent references. Thus, the aim of this study is to conduct a bibliometric analysis of publications on pediatric mycoplasma pneumonia from 2011 to 2023, shedding light on the current state of research and emerging themes in the field to guide future research and clinical decisions.

The pathogenesis remains unclear, with diagnostic methods varying across primary care settings, and treatment complexities escalating due to the widespread misuse of macrolide-resistant *M pneumoniae* (MRMP), particularly in China. Therefore, the purpose of this study is to conduct a bibliometric analysis of publications on MPP in children from 2011 to 2023. This analysis aims to identify the current state of research and emerging themes in the field to inform future research and clinical decision-making.

## 2. Materials and methods

### 2.1. Data sources and search strategies

In this study, the Science Citation Index Expanded and Social Sciences Citation Index within Web of Science (WoS), an internationally recognized scientific research database, were utilized to explore *M pneumoniae* pneumonia (MPP) in children. The search employed keywords limited to English, including TS = (“*Mycoplasma pneumoniae* pneumonia” OR “Mycoplasma pneumonia”) AND (“children” OR “child” OR “childhood” OR “pediatric”) AND “Language = English.” To mitigate daily data updates’ bias, the search period spanned from January 1, 2011, to December 20, 2023, yielding 1054 documents. Only original articles and reviews using normal peer review processes were considered eligible. The 993 documents were exported and saved as full records and cited references in the “RefWorks” format. To ensure data consistency and avoid discrepancies due to database updates, all data were gathered within a single day.

**Figure 1.**
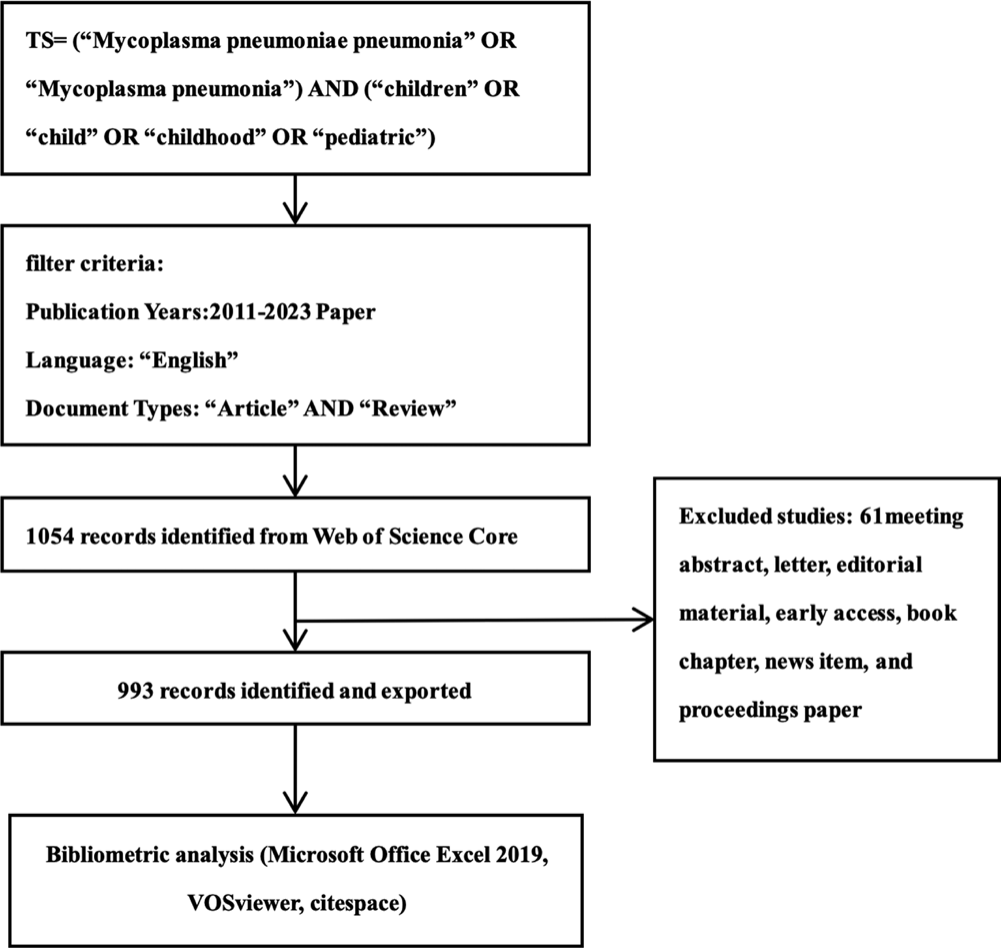
Data filtration flowchart.

Following independent manual screening and discussion among 3 authors to eliminate repetitive, withdrawn, and irrelevant literature, a total of 993 articles on MP pneumonia in children from 2011 to 2023 were published in 338 academic journals by 5062 authors affiliated with 1381 institutions across 75 countries/regions. The specific operational flowchart is depicted in Figure 1.

### 2.2. Data analysis and visualization

VOS viewer 1.6.18, Scimago Graphica 1.0.36, and CiteSpace 6.2.R6 software were employed for data analysis and visualization. CiteSpace’s search parameters included a time slice from January 1, 2011, to December 20, 2023, with 1 year per slice. The g-index and TopN of keywords, references were defaulted. VOS viewer facilitated the visualization of co-authorship among countries/regions, authors, institutions, co-cited references, and keywords. Scimago Graphica 1.0.36 was utilized to construct a map illustrating cooperation relations between countries, providing further insights into international collaboration.

## 3. Result

### 3.1. Annual publications and citations

**Figure 2.**
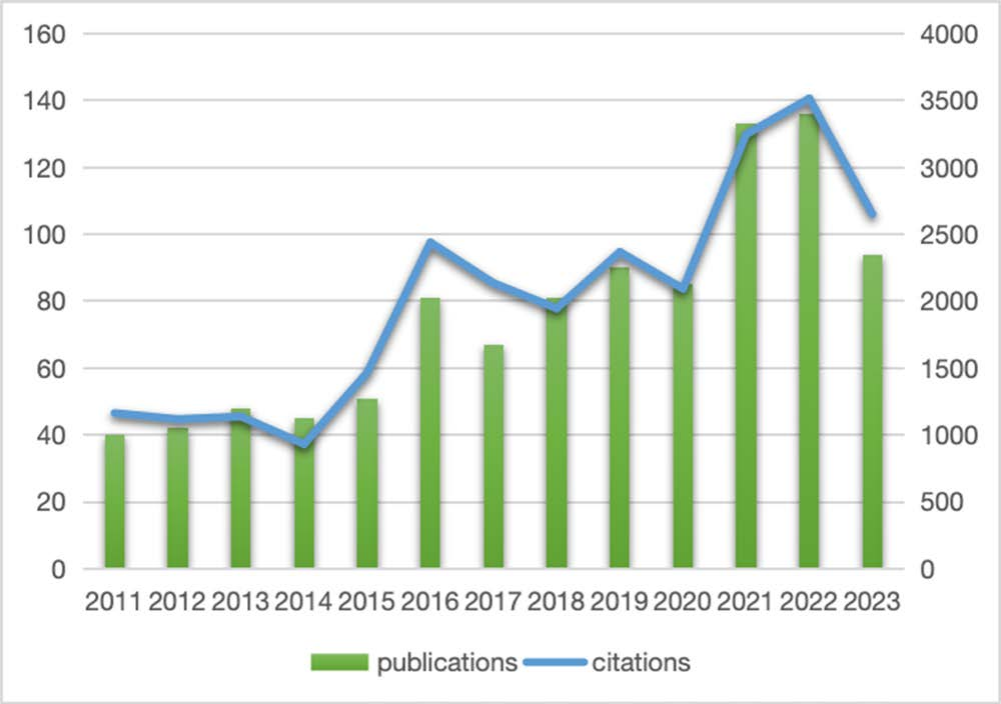
MPP publications and citations over time.

Figure 2 presents the annual count of publications and citations for MPP in children. The number of publications and citations experienced a consistent increase from 2011 to 2022, demonstrating a rapid developmental trend. The zenith occurred in 2021 and 2022, reaching 133 and 136 publications, respectively. By December 20, 2023, an additional 94 papers had been released. Anticipatedly, with the ongoing impact of the epidemic in China during the current autumn and winter, the literature volume is expected to continue its ascent.

### 3.2. Contributions and cooperation among the top 10 producer countries

**Figure 3.**
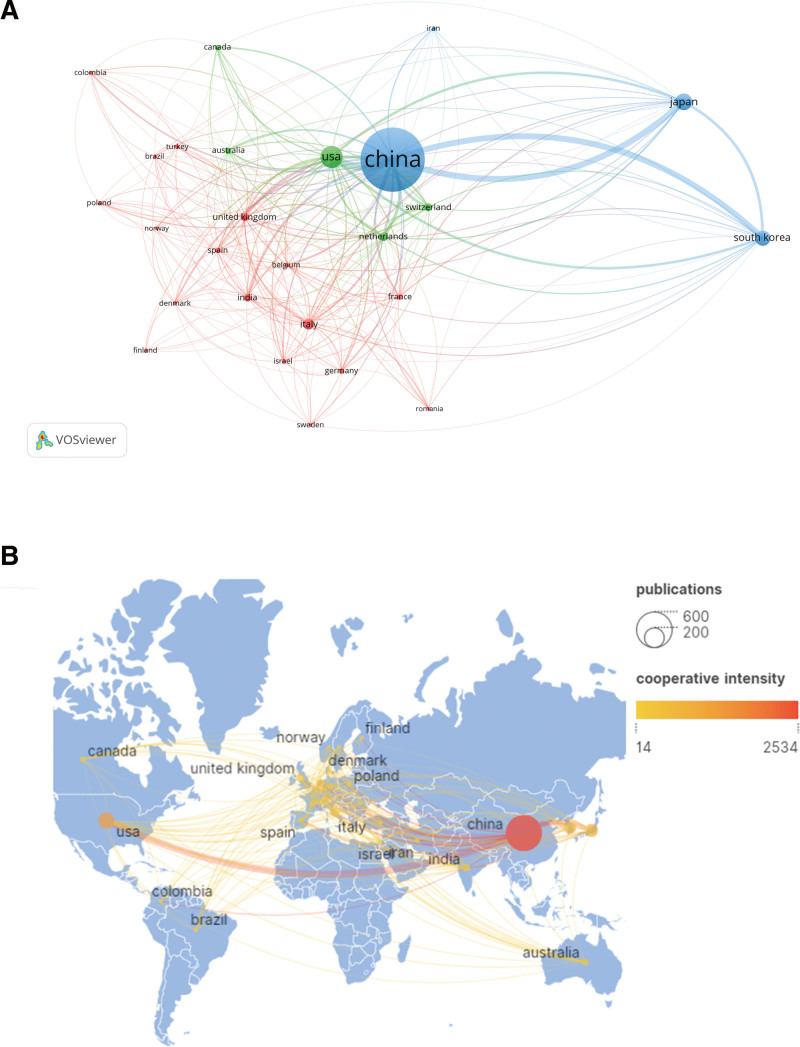
Leading countries/regions. (A) Visual map using VOSviewer network. (B) Distribution of publications and collaborations among countries/regions.

In VOSviewer, 26 countries were identified, with a minimum appearance threshold set to 5. Table 1 outlines the top 10 contributors to these publications. China led with 558 publications (56.19%), followed by the United States (n = 109, 10.97%), and Japan (n = 72, 7.25%). Chinese articles accumulated the highest number of citations (6159 times), while articles from the United Kingdom had the highest average citation rate (68.87 times per article). Figure 3A illustrates the collaborative endeavors of international researchers in conducting studies on MPP in children, with the line thickness denoting the degree of closeness between countries. China emerged as a significant collaborator, engaging extensively with the United States, Japan, Korea, Australia, Switzerland, the Netherlands, and Italy. This collaboration pattern aligns with China’s status as the leading producer of articles and its prominent role in international cooperation.

**Table 1:** The top 10 countries by publications on MPP in children.

Rank	Country	Publications	Total citations	Average citations	Total link strength	Centrality
1	China	558	6159	11.03	2534	0.23
2	United States	109	4091	37.53	1257	0.44
3	Japan	72	1533	21.29	765	0.00
4	South Korea	61	826	13.54	715	0.00
5	Italy	36	728	20.22	233	0.09
6	Netherlands	26	790	30.38	338	0.17
7	Switzerland	24	411	17.12	296	0.04
8	United Kingdom	24	1653	68.87	294	0.21
9	India	21	405	19.28	157	0.08
10	Australia	18	477	26.5	142	0.05

Utilizing Scimago Graphica to generate a visualization map of country cooperation, marks were set to map, and cooperation intensity was represented by orange and red hues. In Figure 3B, the collaboration between China and the United States emerges as the strongest, with varying degrees of cooperation intensity denoted by color.

### 3.3. Authors analysis

**Figure 4.**
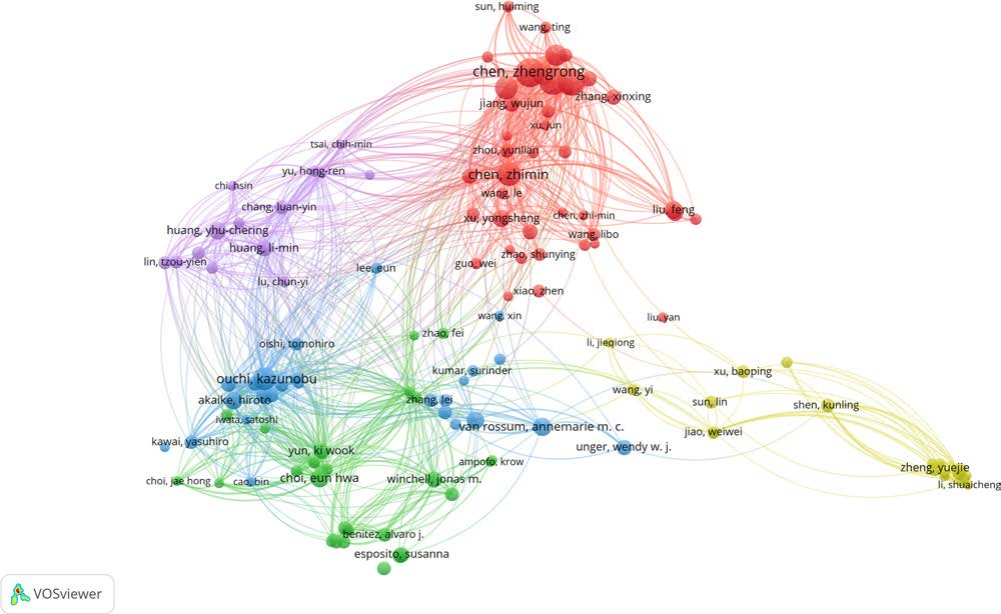
Collaborative network of authors.

Figure 4 illustrates the collaboration networks between authors in the field of MPP in children. Tables 2 and 3 delineate the top 10 authors based on the total citations and most publications, respectively. These tables objectively reflect the researcher’s contribution to the academic field. Notably, Zhengrong Chen from the Children’s Hospital of Soochow University led with the most articles (24 papers), followed closely by Wei Ji and Yongdong Yan from the same hospital, each with 22 papers. Zhimin Chen from Zhejiang University School of Medicine demonstrated the highest H-index at 32. Ouchi kazunobu’s articles received the most citations (424 times), while Yang Liu had the highest average citations (58.66 times). The node diameter represents the productivity of each author, with the lines indicating relationships between authors and the line thickness denoting the intensity of communication. The minimum number of papers per author was set as 5. Of the remaining 127 authors, there were several communities, with each community clustering near 2 or 3 frequently published authors. Connections among various communities were notably limited, suggesting a lack of solid collaboration among research groups/labs engaged in MPP studies.

**Table 2:** The top 10 authors with the most total citations on MPP in children.

Rank	Author	Publications	Total citations	Average citations	Country	Total link strength	H-index
1	Ouchi Kazunobu	17	424	24.94	Japan	527	20
2	Zhimin Chen	18	387	21.5	United States	708	32
3	Wei Ji	22	360	16.36	China	163	26
4	Xiao Li	5	357	71.4	China	97	5
5	Yongdong Yan	22	353	16.04	China	525	18
6	Yang Liu	6	352	58.66	China	414	0
7	Zhengrong Chen	24	347	14.45	China	504	22
8	Akaike Hiroto	11	344	31.27	Japan	94	5
9	Winchell Jonas M.	10	344	34.4	United States	146	25
10	Miyashita Naoyuki	9	327	36.33	Japan	24	21

**Table 3:** The top 10 authors with the most publications on MPP in children.

Rank	Author	Total citations	Publications	Average citations	Country	Total link strength	H-index
1	Zhengrong Chen	24	347	14.45	China	504	22
2	Wei Ji	22	360	16.36	China	481	29
3	Yongdong Yan	22	353	16.04	China	525	18
4	Zhimin Chen	18	387	21.5	United States	708	32
5	Chuangli Hao	17	181	10.64	China	384	14
6	Ouchi Kazunobu	17	424	24.94	Japan	527	20
7	Yuqing Wang	16	251	15.68	China	372	3
8	Huang Li	15	239	15.93	China	448	20
9	Choi Eun Hwa	12	294	24.5	South korea	441	22
10	Van Rossum Annemarie M. C.	12	295	24.58	Netherlands	304	0

### 3.4. Institutions analysis

In VOSviewer, 53 institutions were identified, with a minimum appearance threshold set to 6. Table 4 outlining the top 10 institutions in terms of MPP in children, predominantly universities. Soochow University led with 45 publications, followed by Capital Medical University (n = 44), Zhejiang University (n = 44), Shanghai Jiao Tong University (n = 35), Fudan University(n = 26), and Nanjing Medical University (n = 26). Interestingly, we found that the top 3 authors in terms of the number of papers published were affiliated with the institutions with the highest number of publications, which indicated a high level of contribution and activity of the Soochow University in this area. Among the top 10 institutions, 8 are Chinese, one is South Korean, and one is Japan.

**Table 4:** The top 10 institutions on MPP in children.

Rank	Organization	Publications	Total citations	Average citations	Total link strength	Country
1	Soochow University	45	578	12.84	431	China
2	Capital Medical University	44	633	14.38	507	China
3	Zhejiang University	44	758	17.22	650	China
4	Shanghai Jiao Tong University	35	344	9.82	361	China
5	Fudan University	26	715	27.5	481	China
6	Nanjing Medical University	26	212	8.15	250	China
7	Chang Gung University	23	448	19.47	427	China
8	Seoul National University	18	374	20.77	379	South Korea
9	China Medical University	17	173	10.17	185	China
10	Kawasaki Medical School	17	424	24.94	276	Japan

**Figure 5.**
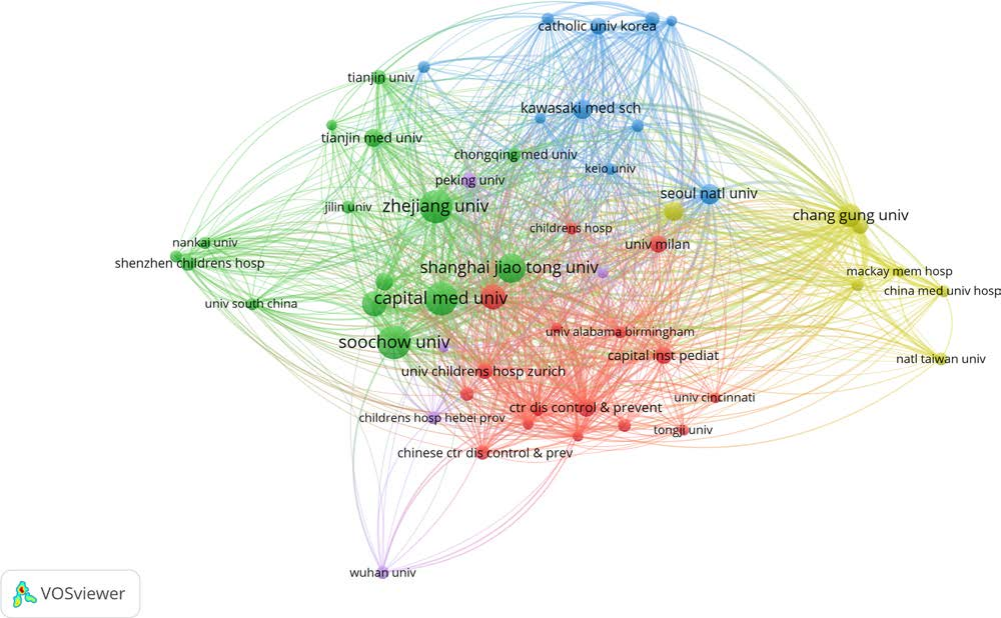
Collaborative network of institutions. (A) Visual map using VOSviewer network among institutions.

Figure 5 illustrates the collaboration networks between institutions in the field of MPP in children. Collaborative efforts among different organizations play a crucial role in mitigating geographic bias and enhancing the reliability of trial results. This collaboration, in turn, provides robust evidence for evidence-based medicine, guiding the development of clinical guidelines. Notably, a robust partnership is evident among various institutions, with a particularly close collaboration observed between China, the United States, Japan, and South Korea.

### 3.5. Analysis of top co-cited references

**Figure 6.**
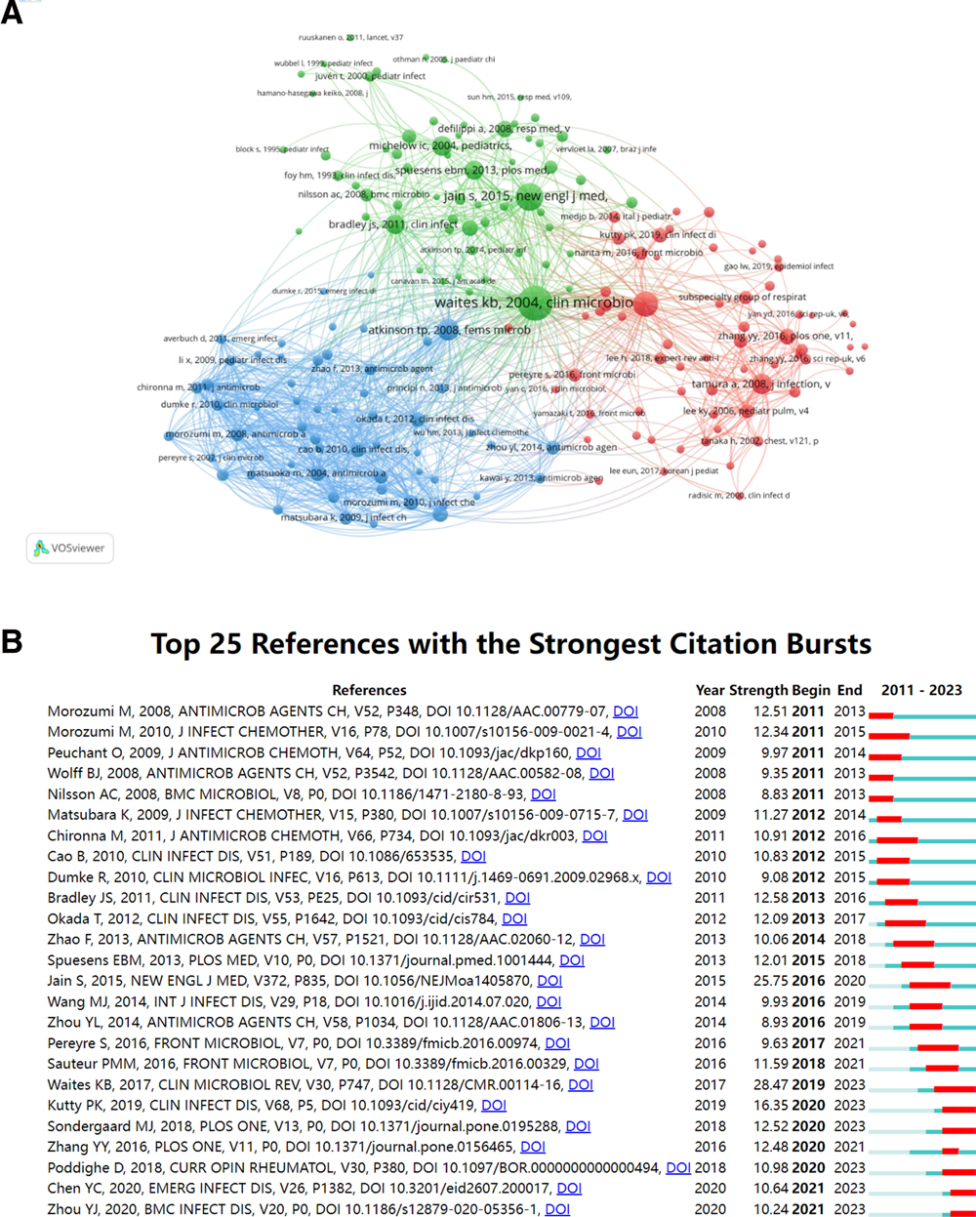
References co-citation network. (A) Knowledge map of co-cited references network. (B) Top 25 references with the strongest citation bursts.

Figure 6A illustrates the co-citation relationships among 180 publications that have been cited no less than 20 times, whereas Table 5 provides insights into the top 10 most co-cited articles in the study of MPP in children. Co-cited literature is a crucial indicator in academic research, reflecting the influence, academic value, and research hotspots of specific publications. Figure 6B displays the top 25 references with the strongest citation bursts. The blue line represents the observed time interval from 2011 to 2023, while the red line represents the burst duration. Several articles, including “*Mycoplasma pneumoniae* from the Respiratory Tract and Beyond,” “*Mycoplasma pneumoniae* Among Children Hospitalized With Community-acquired Pneumonia,” “Clinical manifestations in infants and children with *M pneumoniae* infection,” “Macrolide-Resistant *M pneumoniae* Infections in Pediatric Community-Acquired Pneumonia” “Impact of viral coinfection and macrolide-resistant mycoplasma infection in children with refractory *M pneumoniae* pneumonia,” and “Extra-pulmonary diseases related to *M pneumoniae* in children: recent insights into the pathogenesis.” show continuous citation bursts. This suggests that these topics remain research hotspots and may represent potential frontiers in the field of MPP in children. The paper with the strongest burstiness (strength = 28.47) was entitled “*Mycoplasma pneumoniae* from the Respiratory Tract and Beyond,” published by Clin Microbiol Rev in 2017, with citation bursts from 2019 to 2023.

**Table 5:** The top 10 cited references of publications on MPP in children.

Rank	Frequency	Centrality	Title	Journal	Author	Year
1	112	0.16	*Mycoplasma pneumoniae* from the Respiratory Tract and Beyond^[6]^	*Clin Microbiol Rev*	Waites KB	2017
2	89	0.13	Community-acquired pneumonia requiring hospitalization among U.S. children^[3]^	*N Engl J Med*	Jain S	2015
3	66	0.10	*Mycoplasma pneumoniae* Among Children Hospitalized with Community-acquired Pneumonia^[7]^	*Clin Infect Dis*	Kutty PK	2019
4	49	0.12	Antimicrobial therapy of macrolide-resistant *Mycoplasma pneumoniae* pneumonia in children^[11]^	*Expert Rev Anti Infect Ther*	Lee H	2018
5	45	0.03	The Clinical Characteristics and Predictors of Refractory *Mycoplasma pneumoniae* Pneumonia in Children^[12]^	*PLoS One*	Zhang YY	2016
6	39	0.08	Infection with and Carriage of *Mycoplasma pneumoniae* in Children^[13]^	*Front Microbiol*	Sauteur PMM	2016
7	35	0.02	Clinical manifestations in infants and children with *Mycoplasma pneumoniae* infection^[14]^	*PLoS One*	Sondergaard MJ	2018
8	35	0.01	The epidemiology of pediatric *Mycoplasma pneumoniae* pneumonia in North China: 2006 to 2016^[15]^	*Epidemiol Infect*	Gao LW	2019
9	33	0.02	*Mycoplasma pneumoniae*: Current Knowledge on Macrolide Resistance and Treatment^[16]^	*Front Microbiol*	Pereyre S	2016
10	32	0.16	Rapid effectiveness of minocycline or doxycycline against macrolide-resistant *Mycoplasma pneumoniae* infection in a 2011 outbreak among Japanese children^[17]^	*Clin Infect Dis*	Waites KB	2017

### 3.6. Analysis of hotspots and frontiers

The analysis of co-occurring keywords through knowledge map analysis is a crucial aspect of bibliometrics research, providing clear insights into primary directions and research hotspots in a specific domain. In the co-occurrence analysis using VOSviewer, 52 keywords were identified, with a minimum appearance threshold set to 15. Table 6 presents the most frequent keywords in this study, including “*Mycoplasma pneumoniae* pneumonia,” “child” “infections,” “community-acquired pneumonia,” “pneumonia,” “epidemiology,” and “real-time pcr.” Additionally, other top-ranking keywords like “macrolide resistance,” “pathogenesis,” and “azithromycin” reflect the current hotspots in the field.

**Table 6:** The top 20 keywords on the research of MPP in children.

Rank	Keyword	Occurrences	Total link strength	Rank	Keyword	Occurrences	Total link strength
1	mycoplasma pneumoniae pneumonia	577	2392	11	pathogenesis	80	443
2	child	550	2395	12	etiology	68	359
3	infections	332	1557	13	Chlamydophila pneumoniae	61	339
4	community-acquired pneumonia	290	1550	14	virus	58	294
5	pneumonia	169	832	15	assay	56	325
6	epidemiology	148	806	16	Streptococcus pneumoniae	54	289
7	real-time PCR	144	831	17	strains	50	288
8	diagnosis	139	658	18	disease	48	227
9	macrolide resistance	98	548	19	therapy	47	228
10	clinical characteristics	84	448	20	azithromycin	44	161

**Figure 7.**
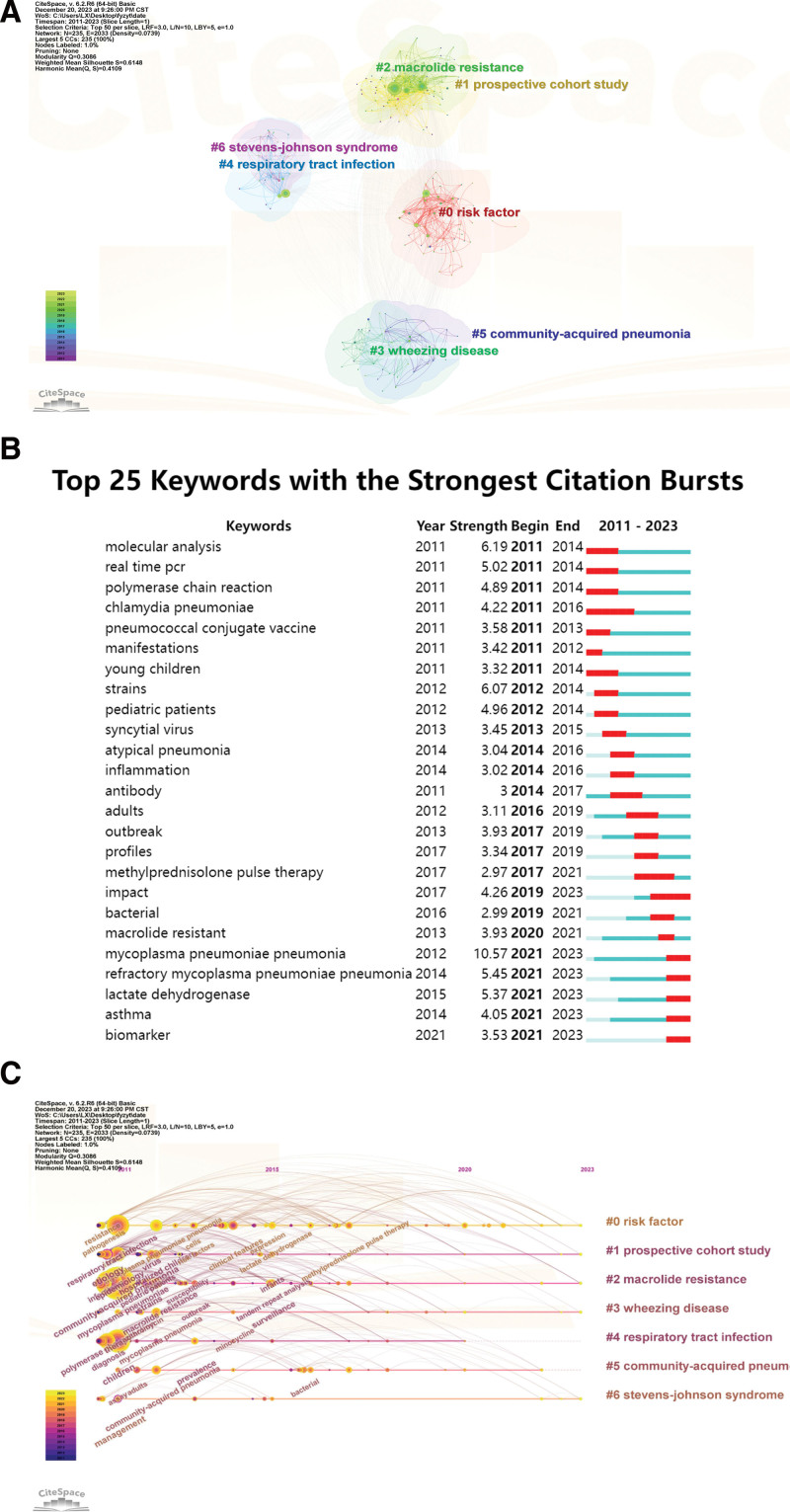
Keywords visualization on MPP in children. (A) Keywords co-occurrence network. (B) Top 25 keywords with the strongest citation bursts. (C) The timeline view of the keywords co-citation network.

As depicted in Figure 7B, keywords with high burst intensity play a crucial role in identifying what is currently hot, cutting-edge, and the latest trend in research. The analysis highlights the top 25 keywords with the strongest burst strength. Notably, keywords such as “*Mycoplasma pneumoniae* pneumonia,” “refractory *Mycoplasma pneumoniae* pneumonia,” “lactate dehydrogenase,” “asthma,” and “biomarker.” continue to experience citation explosions until 2023. This suggests that these research directions have gained increased attention from clinicians and academics over the last 2 years and are likely to become prominent research topics in the future. The timeline view of the clustering plot is shown in Figure 7C, which aids identification of emerging research hotspots of MPP in children. Utilizing CiteSpace parameters, a network of 235 nodes with 2033 connections was generated (Fig. 7A). Term co-occurrence analysis revealed 7 primary clusters represented by different colors, including “#0 risk factor,” “#1 prospective cohort study,” “#2 macrolide resistance,” “#3 wheezing disease,” “#4 respiratory tract infection,” “#5 community-acquired pneumonia,” and “#6 stevens-johnson syndrome.”

The “#0 risk factor” cluster mainly includes infection, resistance, clinical features, pathogenesis, lactate dehydrogenase, plastic bronchitis, immunoglobulin, inflammatory factors, risk factors, cytokines, inflammation, refractory *M pneumoniae* pneumonia, bronchoalveolar lavage procedures, fiberoptic bronchoscopy, lavage bronchoalveolar, biomarker, nomogram model, vitamin A, chemokine, CARDS toxin, and Th 17.

The “#1 prospective cohort study” cluster mainly includes transmission, epidemiology, diagnostic, tetracyclines, quinolones, glycylcycline tigecycline, macrolides, pneumococcal conjugate vaccine, laboratory techniques, *M pneumoniae* igm, *M pneumoniae* iga, real-time polymerase chain reaction, multiplex polymerase chain reaction, virus, community-acquired pneumonia, severe pneumonia, vaccine, and identification.

The “#2 macrolide resistance” cluster mainly includes metagenomic next-generation sequencing, macrolide resistance, 23S rRNA, epidemiology, Polymerase Chain Reaction (PCR), pathogens, risk factors, pneumonia, antibody, assays, severe *M pneumoniae* pneumonia, seasonality, serological tests, lower respiratory tract infection, tract infection, cytokines, and lactate dehydrogenase.

The “#3 wheezing disease” cluster mainly includes repeated wheezing, asthma, gut microbiota, bacteria, azithromycin, minocycline, and efficacy.

The “#4 respiratory tract infection” cluster mainly includes respiratory syncytial virus, human bocavirus, bacterial pathogens, human adenovirus, respiratory infection, virus, identification, viral infection, respiratory virus, parainfluenza viruses, respiratory pathogens, antibiotic, influenza virus, respiratory microbiome chlamydia pneumoniae, and lactate dehydrogenase.

The “#5 community-acquired pneumonia” cluster mainly includes community-acquired pneumonia, lung, culture, serological assays,activation, acute exacerbation, c-reactive protein, multiplex polymerase chain reaction, procalcitoninconjugate vaccine, procalcitonin, assay, bacterial and severity.

The “#6 stevens-johnson syndrome” cluster mainly includes management,risk, erythema multiforme, stevens-johnson syndrome, toxic epidermal necrolysis, classification, antinuclear antibodies.

## 4. Discussion

### 4.1. General information

Analysis of data from the WoSCC database covering the period 2011 to 2023 reveals a total of 993 publications on MPP in children. These were distributed across 338 academic journals, involving 5062 authors affiliated with 1381 institutions spanning 75 countries/regions. A consistent upward trend in global publications is observed, peaking at 136 in 2022. Although 2023 data is incomplete, it is anticipated that the literature output will continue to rise, likely influenced by the recent epidemic outbreak in China. Turning points in annual growth and total annual citations occurred in 2016 and 2021. MPP may manifest in regional epidemics every 3 to 7 years, lasting up to 2 years. Previous research indicates a global surge in MPP cases among children during 2015 to 2016.^[11,12,18]^ A noteworthy finding from a study involving 34 research centers in 20 countries reveals a substantial decline in MPP incidence among children following the implementation of preventive measures (e.g., mask-wearing and social activity restrictions) employed to control COVID-19. Equally noteworthy is the sustained reduction in MPP cases among children in 2021 to 2022, despite the easing of social restrictions during this period.^[13]^ Literature on RMMP, macrolide-resistant *M pneumoniae* (MRMP), and Severe MPP (SMPP) has surged in the past 2 years. This surge suggests an alarming rise in children exhibiting macrolide resistance, leading to co-infections and the development of RMMP and SMPP. This trend is raising concerns among researchers and clinicians.

According to Table 1 and Figure 1, China dominates the MPP research landscape, contributing 56.19% (558 publications) of the total publications and citations. China also leads in collaborative networks, particularly with the United States, Japan, Korea, India, and the Netherlands. Despite China’s numerical dominance, the United Kingdom maintains a superior position in terms of average article citations. Among the top 10 institutions contributing to MPP publications, 8 are Chinese, one is South Korean, and one is Japan. Soochow University led with 45 publications, followed by Capital Medical University and Zhejiang University (n = 44). A close interconnection is observed among various countries and institutions. Fostering robust collaboration and dialogue between nations and organizations is crucial to overcoming academic barriers and promoting advancements in MPP-related research.

Zhengrong Chen, with 24 publications, stands out as the foremost author in *M pneumoniae* research, followed closely by Wei Ji and Yongdong Yan, each with 22 publications. This underscores their significant contributions to the field. Zhimin Chen from Zhejiang University School of Medicine boasts the highest h-index, a comprehensive quantitative metric reflecting a researcher’s scholarly output and academic impact.^[14]^ Zhimin Chen focuses on the clinical characteristics and risk factors of *M pneumoniae* pneumonia.^[15,16]^

Co-cited literature serves as a crucial indicator in academic research, reflecting the influence, academic value, and research focus of specific works.^[17]^ The most-cited publication, a 2017 article in Clin Microbiol Rev by Waites KB et al, titled “*Mycoplasma pneumoniae* from the Respiratory Tract and Beyond,” boasts 112 citations and exhibits the most robust citation burst during 2019 to 2023.^[9]^ In this study, Waites KB et al provide a comprehensive review of recent developments in MP, including the publication of full genome sequences of additional strains, offering deeper insights into pathogenic mechanisms. Notably, there is a global emergence of clinically significant acquired macrolide resistance, leading to the standardization of in vitro susceptibility testing methodologies. Additionally, several new drugs effective against MP are in development.

### 4.2. Hotspots and frontiers

The utilization of keyword clustering can unveil the underlying research structure in the field of MPP. Through careful examination of these analyses, a wealth of valuable information can be gleaned, including but not limited to risk factor, prospective cohort study, macrolide resistance, wheezing disease, respiratory tract infection, community-acquired pneumonia, and Stevens-Johnson syndrome. In subsequent sections we will discuss their profound significance for this area of research as well as potential implications for future directions.

#### 4.2.1. Analysis of risk factor.

Plastic bronchitis (PB) as an acute and critical lung disease is an important factor to make MPP refractory.^[19]^ Fiberoptic bronchoscopy (FOB) and bronchoalveolar lavage procedures (BAL) are effective in the management of PB, so early identification of patients at high risk for PB in children with Refractory MPP (RMPP) is important. In the recent 2 years, as a new type of predictive model with easy clinical application, strong discriminatory ability and high accuracy, the nomogram model has been confirmed in several retrospective studies for its validity as an early identification of RMPP.^[19–23]^ However, the variability of the indicators included in different studies has led to a lack of consistency in the nomogram model, and external validation by prospective multicenter studies is needed for its clinical application.

Recent studies reveal a negative correlation between serum vitamin A levels and the severity of MPP, suggesting adequate serum vitamin A as an independent protective factor against RMPP. This protective effect may be attributed to vitamin A’s pleiotropic role in maintaining a normal mucosal barrier, preventing invasive pathogens, enhancing lung immune function, and modulating inflammation.^[24]^ Inflammatory cytokines (IL-6, IL-18, and IL-10), D-dimer, C-reactive protein (CRP), and lactate dehydrogenase have proven to be reliable predictors of RMPP in several studies.^[25–28]^

The pro-inflammatory chemokine CXCL10 may serve as a potential predictor of SMPP in children. The mechanism could be the development of autoimmune inflammation through antigenic cross-reactivity, leading to autoimmune diseases in multiple organ systems.^[29]^

Expression levels of CARDS toxin and the inflammatory factor IFN-γ/CXCL9 positively correlate with the severity of MPP, emerging as new biological indicators for early prediction and clinical detection of MPP.^[30,31]^ Recent research suggests that CARDS toxin stimulates a Th1-type immune-inflammatory reaction in the lungs. Subsequently, the JAK/STAT 1 signaling pathway, activated by IFN-γ, promotes CXCL9 secretion. CXCL9 facilitates the movement of more Th1 cells to the center of the inflammatory response, creating a positive feedback loop that amplifies the inflammatory cascade and exacerbates immunity-related injury in lung tissue.^[32]^

Recent research has shown that the co-stimulatory molecule B7-H3 played an important role in the immune-inflammatory response of MPP through the over-regulation of Th 17 differentiation and the enhancement of IL-17 secretion. sB 7-H3 and IL-17 levels were elevated in the peripheral blood of children with MPP, while the expression of miR-29 c was lowered, and the expression of sB 7-H3 and IL-17 were positively correlated. Targeting miR-29c and B7-H3 could be a novel approach in preventing and treating MPP, potentially serving as innovative biomarkers for prognostic evaluation.^[33]^

#### 4.2.2. Analysis of prospective cohort study and community-acquired pneumonia.

Since these 2 keyword clusters have a high degree of overlap, we will analyze and discuss them in the same section.

CAP is a pulmonary infection affecting the parenchyma or pleura, contracted outside a hospital setting, which poses a significant global risk to public health.^[34]^ MP is a significant contributor to CAP, with a global incidence in pediatric patients ranging from 10% to 40%.^[35,36]^ However, during epidemics, this organism is responsible for 20% to 40% of CAP cases in the general populace, escalating to 70% in closed populations.^[37,38]^

The substantial impact of MPP on children’s health emphasizes the need for accurate MPP case recognition and prevalence reduction, which relies heavily on precise diagnostic techniques.

While most MPP cases are mild and self-limiting, the incidence of RMPP and SMPP is increasing due to rising pathogen resistance and an uptick in co-infections in recent years. Some children experience severe intrapulmonary and extrapulmonary complications.^[39]^

Despite the increased resistance of MP to macrolides in recent years, macrolides are still the preferred drugs for the clinical treatment of pediatric MPP, such as azithromycin. The tetracyclines, quinolones, and glycylcycline tigecycline are alternatives for the treatment of MRMP, but all have different adverse effects. With the growth and global spread of macrolide resistance in MP, the development of new drugs to prevent infection or improve symptoms is of increasing interest. The solithromycin is a 4th generation macrolide antimicrobial in development, belonging to the ketolides, and it is the most effective antimicrobial agent tested against MP to date.^[40]^ Lefamulin, also known as BC-3781, a recently approved novel antibacterial, is accessible in both intravenous and oral forms, showing strong efficacy against numerous Gram-positive and Gram-negative bacteria and is presently under clinical trials for CAP therapy.^[41]^ In the published prescribing information for lefamulin, there are gaps in the data on the safety and efficacy of the drug in children. A current report showed that anticancer and anticancer and antiviral nucleoside and nucleobase analogs inhibit MP growth, demonstrating that enzymes involved in nucleotide biosynthesis are potential future targets for new drugs in the treatment of MPP.^[42]^

Current laboratory techniques for detecting MP infection include culture, serological assays, and nucleic acid amplification tests. However, each method has limitations. Culture, while the gold standard, is hindered by long processing times, additional species identification procedures, and low sensitivity, limiting its routine clinical application.^[43]^ Serological tests targeting immunoglobulin A, immunoglobulin G, and immunoglobulin M may yield false results, as antibodies can persist post-infection, potentially leading to unnecessary antibiotic use.^[44]^ Infants’ underdeveloped immune systems and potential cross-reactivity with other pathogens may result in false-negative or false-positive serum antibodies.^[45,46]^ According to the Chinese Guidelines for the Diagnosis and Treatment of *M pneumoniae* Pneumonia in Children (2023 edition), an accurate serologic diagnosis requires a single serum MP antibody titer ≥ 1:160 or a 4-fold increase in MP antibody titer in 2 sera (at least 2 weeks apart) during the course of the disease.^[47]^ However, the logistical challenge of repeated blood collection over 2 weeks and the demonstrated missed diagnosis rates for MP antibody titers ≥ 1:160 pose significant challenges in clinical settings. PCR, with its various methods such as routine, nested, real-time, multiplex, and isothermal amplification, is considered the new gold standard for its high sensitivity and specificity. However, it cannot distinguish between MP colonization and true infection.^[48–51]^ The Loop-Mediated Isothermal Amplification technique, valued for its sensitivity, specificity, rapidity, and simplicity, is now the preferred diagnostic method for acute MP diagnosis in Japan. A novel Loop-Mediated Isothermal Amplification-LFB assay targeting Community-Acquired Respiratory Distress Syndrome (CARDS) toxin genes, boasting a 2-minute detection time, requires no additional instrumentation, and exhibits high sensitivity, is poised for widespread clinical use.

In recent years, various serum molecular markers, including procalcitonin and CRP, have been developed to assess the severity of Community-Acquired Pneumonia (CAP) in children. The use of an elevated CRP to procalcitonin ratio as a predictor for *M pneumonia* in adults has been explored, but evidence in pediatric cases is still limited.^[52–54]^

Following the widespread use of pneumococcal conjugate vaccines, *M pneumoniae* has emerged as the predominant bacterial culprit in lower respiratory tract infections in children.^[55]^ Currently, there is no vaccine available to prevent *M pneumoniae* infections, but a variety of MP vaccines are under development, including whole-cell vaccines (inactivated and live-attenuated vaccines), subunit vaccines (involving *M pneumoniae* protein P1, protein P30, protein P116 and CARDS toxin) and DNA vaccines.^[56,57]^ live vector vaccines have unique advantages, such as high safety, can induce humoral and mucosal immunity for a long period of time, and be produced as a multivalent vaccine, which makes it a potential vaccine candidate and one of the most promising genetically engineered vaccines at present.^[58]^ Single-antigen protein formulated with vaccine adjuvant and multi-epitope fusion protein rank high as potential candidates for vaccines.^[57]^

#### 4.2.3. Analysis of macrolide resistance.

Macrolides, serving as primary drugs for treating MPP and commonly administered in outpatient settings, face a rising epidemic of MRMP worldwide, particularly in East Asia.^[19]^ MRMP rates are strikingly high, reaching 90% to 100% in China, 87% in Japan, and 84.6% in Korea. Substantial increases are also observed in Italy (26%), Scotland (19%), and the United States (13%).^[11,59,60]^ The prevalence of MRMP in East Asia significantly differs from that reported in North America or Europe.

An increasing number of reports employ diverse molecular techniques to understand the dissemination of MRMP in Asia. Some studies, utilizing multilogues variable-number tandem-repeat analysis as the molecular typing method, suggest a polyclonal dissemination of *M pneumoniae*.^[61,62]^ However, recent reports from South Korea and Japan, employing multilogue sequence typing as the diagnostic technique, reveal a correlation between the extensive dissemination of MRMP and the clonal expansion of the resistant ST3 clone.^[63,64]^ Future investigations may benefit from whole-genome sequencing as a more effective and comprehensive approach to address discrepancies and explore the evolutionary patterns of MP.

The reduced efficacy of macrolides in treating MRMP prompts ongoing debate regarding whether resistant strains lead to more severe illnesses. Numerous studies suggest that MRMP in children is associated with increased instances of refractory *M pneumoniae* pneumonia (RMPP), extrapulmonary complications (encephalitis, myocarditis, or hepatitis), and more severe clinical symptoms and radiological signs.^[65,66]^ Conversely, randomized studies indicate that approximately 30% of patients infected with macrolide-resistant strains can still be effectively treated with macrolides.^[67,68]^ While MP infection is typically self-restricted, the use of ineffective antimicrobial agents leads to delayed treatment and a heightened risk of persistent fever and serious complications. Therefore, prompt diagnosis following disease onset is crucial for preventing deterioration.

Commonly employed molecular biology methods aid in identifying MRMP strains.^[15]^ Research indicates that MRMP exhibits specific mutations in the peptidyl transferase loop of 23S rRNA, along with insertions or deletions in ribosomal proteins L4 and L22.^[46]^ In China, primary MRMP sources are variants in the 23S rRNA gene’s domain V, predominantly featuring an A2063G mutation, where P1 type 1 and type 2 lineages co-circulate.^[65]^

Compared with traditional pathogenicity testing, metagenomic next-generation sequencing (mNGS) as a new molecular diagnostic technology, can detect multiple pathogens at the same time, shorten the detection time, clarify the cause of infection as early as a possible, and detect the drug resistance genes of pathogens, thus avoiding the misuse of antibiotics. However, limitations such as the interference of nucleic acids in the host background and the expensive cost have led to a great limitation of the application in the clinical setting.

Currently, commercial PCR kits are available, facilitating point-of-care identification of MP genes, antigens, and drug resistance mutations concurrently, streamlining MRMP diagnosis.^[69–71]^

Feng Huang et al have identified Circular RNAs as novel Long non-coding RNAs that could serve as valuable biomarkers for early RMPP detection^.[72]^

MRMP poses challenges for pediatricians, especially in pandemic areas. Tetracyclines (TCs) and fluoroquinolones (FQs) are recommended as viable substitutes.^[73]^ However, TCs are contraindicated in children under 8 due to detrimental effects on bone development, tooth enamel hypoplasia, and permanent tooth discoloration.^[39]^ Doxycycline, a second-generation TC with a broad therapeutic spectrum, is recommended by the U.S. Centers for Disease Control and Prevention as an alternative drug for MRMP in children under 8 due to its lower adverse effects.^[43]^ While FQs are generally well-tolerated, long-term treatment poses a risk of joint and tendon damage.^[44,45]^ Therefore, FQ use is generally limited to patients with complicated infections or those without suitable alternative antibiotics, considering the risks of tendinopathy and other adverse events (e.g., peripheral neuropathy and exacerbation of myasthenia gravis).^[61]^

#### 4.2.4. Analysis of wheezing disease.

A significant number of children suffering from MPP experience repeated wheezing and diminished small airway functionality once their clinical signs subside, which often results in asthma.^[74]^ The pathogenesis of MP in asthma is unclear. However, in recent years, the important role of intestinal microecological changes in lung infections has attracted more and more attention from researchers. Jiang et al^[75]^ found a link between blood inflammatory elements and the presence of *Ruminococcus flavefaciens* and *Clostridium butyricum* in children suffering from MPP, alongside a notable reduction in these bacteria in their intestinal tract, potentially exacerbating the inflammatory response in those with wheezing MPP. Achieving equilibrium in the gut microecology could be a forward-looking approach in preventing asthma and, broadly, respiratory illnesses in children suffering from MPP. A latest study found that azithromycin upregulated the expression of IL-10 in the bronchoalveolar lavage fluid of patients with wheezing by inhibiting the NF-κB signaling pathway and downregulation of EZH2-mediated histone H3K27me3 hypermethylation thereby attenuating wheezing following lung inflammation, which may contribute to the development of new approaches for the prevention and treatment of wheezing caused by inflammatory lung diseases in children.^[76]^

#### 4.2.5. Analysis of respiratory tract infection.

Recent reports have demonstrated that the imbalanced respiratory microbiome is associated with the severity of acute respiratory infections, such as MPP.^[77–79]^

Reduced alpha diversity and significantly increased *Haemophilus* abundance in the lower respiratory tract (LRT) microbiome of children with severe MPP compared to those with mild MPP. The abundance of MP aligned with the disease’s intensity, encompassing the complications and distinct blood markers of organ harm.^[80]^

The 16S rRNA gene sequencing has a potential diagnostic role in analyzing respiratory specimens for infectious diseases. However, 16S rRNA gene profiles do not reflect the activity of the microbiota and antibiotic treatment often affects the diversity of the microbiota, so larger datasets will be needed in the future to further investigate the role of live and dead bacteria in inducing inflammation in infectious diseases, and consideration should also be given to assessing the degree to which changes in respiratory microbial community structure over time or with antibiotic treatment affect the MPP.^[81]^

The most common pathogen among co-infected children with MPP was Chlamydia pneumoniae, and the predominant co-infecting bacterium was *S pneumoniae*, followed by *H influenzae* and *Staphylococcus aureus*. Human Bocavirus (HBV), human Rhinovirus, and syncytial virus were the most common sources of viral co-infections.^[82,83]^ However, controversy exists regarding whether viral coinfection is the cause of Refractory MPP in children, and there is no international consensus on RMPP treatment options.

A retrospective study found that children with MPP co-infected with human adenovirus (HAdV) experienced longer hospital stays and fever, higher rates of respiratory distress, oxygen therapy, and noninvasive continuous positive airway pressure use, along with higher rates of severe pneumonia.^[84]^ Previous studies have reported elevated serum lactate dehydrogenase levels in patients with refractory MPP, including children requiring steroid therapy, and this elevation has been observed in children with MPP combined with HAdV.^[85–87]^

This underscores the need for clinicians managing MPP to be vigilant about the potential co-infection with HAdV in the presence of respiratory distress. Timely administration of oxygen therapy and isolation of HAdV-infected children are crucial to prevent serious complications associated with nosocomial infections in MPP-afflicted children. Several Chinese studies have indicated a high detection rate of MPP co-infections with Parainfluenza viruses and Influenza virus. However, clinical symptoms in children with MPP co-infection were statistically indistinguishable from those with MPP infection alone.^[88,89]^ Children with RMPP and co-infections exhibited a prolonged febrile course, elevated white blood cell count, C-reactive protein, and a higher prevalence of pneumothorax or diffuse massive lung inflammation.

Discrepancies between studies highlight the impact of co-infection with different viral infections on the outcome of MPP infection and the clinical manifestations of MPP in children. However, the majority of these studies are retrospective, with either small sample sizes or significant differences in detection rates of viral pathogens due to variations in testing methods. Therefore, there is an imperative need for multicenter, large-sample prospective studies on MPP co-infection with specific viruses to elucidate the relationship between RMPP and viral co-infections. Such studies can provide reliable evidence-based medical insights for clinical guidelines on RMPP in children.

#### 4.2.6. Analysis of Stevens-Johnson syndrome.

In 2015, Canavan et al described *M pneumoniae*-induced rash and mucositis (MIRM) as a rare disease with pathogenesis and clinical manifestations different from erythema multiforme, Stevens-Johnson syndrome (SJS), and toxic epidermal necrolysis (TEN).^[90]^ Due to reports of MIRM are relatively rare, there are no evidence-based medical guidelines to guide clinicians in the management of MIRM.^[90–93]^ The pathogenesis of SJS is unclear, and it may be that polyclonal B cells proliferate and produce specific antibodies and immune complexes after MP infection and are deposited in the skin, after which stimulated cytotoxic T-cells induce skin injury.^[94–96]^ Typically, the progression of MIRM is milder compared to drug-related SJS-TEN, making supportive care a viable choice. Nonetheless, vigilant observation of mucous membranes is crucial due to the occurrence and severity of the same side effects observed in SJS-TEN. Research indicates that the total IgE levels in the serum of patients exhibiting MIRM symptoms surpass those in children with only MPP, which may be related to an immune imbalance.^[97]^ A study found higher antinuclear antibodies titers in patients with MIRM, suggesting that MIRM may predispose to inflammatory autoimmune diseases.^[98]^ Therefore, anti-inflammatory immunosuppressive therapy should be considered, especially early in the acute phase.

## 5. Strengths and limitations.

Bibliometric visualization analysis surpasses conventional literature reviews in terms of intuitiveness and thoroughness.^[99]^ As far as we know, this research is the first comprehensive bibliometric analysis of related literature in the field of *M pneumoniae* pneumonia in children. By analyzing the highly cited literature, reference outbreaks, keyword co-occurrence, clustering and outbreaks, the knowledge structure of this research field was described, and the research frontiers and hot directions of MG research were explored.^[100]^ However, this research has its own set of constraints. Firstly, given the WoSCC database’s status as the predominant resource in bibliometric studies,^[101,102]^ our analysis was limited to the WoSCC database and enforced specific limitations on the types of languages and documents, which may have resulted in the exclusion of pertinent literature from alternative sources.^[103]^ Additionally, owing to the ongoing refreshment of the database, the dataset for this year remains unfinished, and the latest high-quality literature was excluded from this research. Thirdly, due to the time-dependent nature of citation metrics, bibliometrics are unable to evaluate the level of individual studies, suggesting that newer articles might receive less citations than their predecessors, primarily because of their publication dates.

## 6. Conclusion

By analyzing countries, institutions, original articles, authors, co-cited references, and keywords, this study has delineated the knowledge base of *M pneumoniae* pneumonia in children research findings over the past 13 years. It reveals future trends, offering researchers and clinicians valuable insights for research directions and clinical decisions.

## Author contributions

**Conceptualization:** Congcong Liu.

**Data curation:** Congcong Liu, Rui Wang, Shuyi Ge, Binding Wang, Siman Li.

**Formal analysis:** Congcong Liu, Rui Wang, Shuyi Ge, Binding Wang, Siman Li.

**Visualization:** Congcong Liu, Rui Wang, Shuyi Ge, Binding Wang, Siman Li.

**Writing – original draft:** Congcong Liu.

**Writing – review & editing:** Congcong Liu, Bohua Yan.
